# Colorectal cancer disparities in Latin America: Mortality trends 1990–2019 and a paradox association with human development

**DOI:** 10.1371/journal.pone.0289675

**Published:** 2023-08-25

**Authors:** Camila D. Muzi, Matthew P. Banegas, Raphael M. Guimarães

**Affiliations:** 1 Brazilian National Cancer Institute, Rio de Janeiro, Brazil; 2 University of California, San Diego, La Jolla, CA, United States of America; 3 National School of Public Health, Oswaldo Cruz Foundation, Rio de Janeiro, Brazil; Massachusetts General Hospital, UNITED STATES

## Abstract

**Background:**

Colorectal cancer mortality is growing in Latin America. It is known for a marked income disparity between its countries, and there is a consistent association with development. Our purpose was to describe trends in colorectal cancer mortality in Latin America between 1990 and 2019, identifying differences by human development categories.

**Methods:**

We extracted age-adjusted mortality rate from the Global Burden of Disease (GBD) Study from 22 Latin American countries, subregions, and country groups previously ranked by the GBD study due to Sociodemographic Index (SDI) between 1990 and 2019. We applied the segmented regression model to analyze the time trend. Also, we estimated the correlation between mortality rates and Human Development Index (HDI) categories for countries.

**Results:**

Between 1990 and 2019, colorectal cancer adjusted mortality rate increased by 20.56% in Latin America (95% CI 19.75% - 21.25%). Between 1990 and 2004, the average annual percentage change (APC) was 0.11% per year (95% CI 0.10–0.12), and between 2004 and 2019 there was a deceleration (APC = 0.04% per year, 95% CI 0.03%– 0.05%). There is great heterogeneity among the countries of the region. Correlation between these two variables was 0.52 for 1990 and 2019. When separated into HDI groups, the correlation varied in the direction of the association and its magnitude, typifying an effect modification known as Simpson’s Paradox.

**Conclusions:**

Human development factors may be important for assessing variation in cancer mortality on a global scale. Studies that assess the social and -economic contexts of countries are necessary for robust evaluation and provision of preventive, diagnostic and curative services to reduce cancer mortality in Latin America.

## Introduction

Cancer is one of the most significant barriers to increasing life expectancy and is responsible for most premature deaths worldwide [1]. Colorectal cancer ranks second among cancer sites with the highest mortality. GLOBOCAN estimates more than 900,000 deaths in 2020, representing 10% of cancer deaths overall [2]. The Global Burden of Disease (GBD) study estimated that the burden of illness associated with colon and rectal cancer caused 24.3 million years of disability worldwide, of which 4.4% are caused by premature death. In 2019, colorectal cancer became the leading cause of death in 9 countries for women and 11 countries for men [3].

Indeed, in Latin America, cancer incidence and mortality have been rising over the last decades [4]. There is a well-known association between cancer incidence and mortality and the socio-economic development of countries [5]. Concerning colorectal cancer, there is a positive correlation between Human Development Index (HDI) and age-adjusted mortality rate among 169 countries enrolled in Global Cancer Project [6]. Most likely, this relationship is influenced by significant risk factors for both incidence and mortality, such as sedentary life, processed food consumption and access to early diagnosis, which are associated with countries’ development, a so-called "western way of life"[7]. Despite these findings, a recent systematic review on the socio-economic determinants of disparities in use of cancer screening services in Latin America found no studies regarding the utilization of colorectal cancer screening [8]. It suggests a knowledge gap about the association between socio-economic determinants and cancer outcomes in the region.

Latin America is known for a marked disparity in income and social vulnerability among its countries, compared to other regions globally. Although there has been some progress in reducing economic inequalities within the region, there is a most remarkable divergence in social indicator performance among Latin American countries compared to heterogeneity in other regions with similar levels of economic development [9].

Reports from the GBD Study encourage health disparities analyses by regions, countries, and subnational levels when data are available. Forecasts for colorectal cancer mortality rates through 2035 are expected to continue decreasing in most countries, including Asia, Europe, North America, and Oceania, except for countries in Latin America and the Caribbean [10]. To understand its evolution from a regional perspective, in comparison to international trends, our objective was to describe trends in colorectal cancer mortality in Latin America between 1990 and 2019, identifying differences by Human Development Index categories [11].

## Methods

### Data sources

The GBD Study estimates the disease burden for a range of causes, risk factors, and covariates such as sex and age for dozens of countries, including subnational analyses. It also produces estimates for groups of countries according to sociodemographic development. Bayesian methods are used for local (e.g., national) data from surveys, information systems and predictions when data are scarce. GBD mortality estimates are generated in 2 primary steps: (1) estimation of the cancer mortality-to-incidence ratio, using a space-time Gaussian process regression approach, (2) application of the Cause of Death Ensemble model (CODEm) that combines data from different sources, such as vital registration systems and cancer registries. For each cancer, sex specific CODEm models generate mortality estimates across locations, years, and age groups. Detailed description of the national level data and methodology used by Global Burden of Disease 2019 Cancer Collaboration is previously published [12]. These GBD results serve as comprehensive and comparable estimate that can inform efforts toward equitable cancer control around the world.

We used data from 22 countries in Latin America, in addition to four sub-regions proposed by the GBD study including: a) Andean Latin America (Bolivia, Ecuador and Peru), b) Central Latin America (Colombia, Costa Rica, El Salvador, Guatemala, Honduras, Mexico, Nicaragua, Panama and Venezuela), c) Tropical Latin America (Brazil, Paraguay), d) South Latin America (Argentina, Chile and Uruguay). The Caribbean subregion was excluded due to data incompleteness; however, individual countries were included: Cuba, Dominican Republic, Guyana, Haiti, and Suriname.

We compare Latin American countries and subregions for both descriptive and time series analysis due to social development. As a reference, we also included five clusters created from the GBD study. The clusters are composed of nations and represented by Sociodemographic Index (SDI). The SDI is an indicator calculated from the dimensions of per capita income, years of schooling, and the fertility rate in women under 25 years old [13].

The diagnostic codes used by the GBD study for colorectal cancer were ICD-9 153 and 154 and ICD-10 C18-20, making it possible to analyze all time series [14]. Two researchers (CDM and RMG) independently extracted data from the GBD 2019 database for the global region and Latin America, including mortality data, with rates adjusted by five-year age groups and standardized by the world population, and their respective confidence intervals (CIs) for all years between 1990 and 2019.

In addition, we obtained the Human Development Index estimates for all countries used in the analysis from the United Nations Development Program (UNDP) Data Center [13]. The Human Development Index (HDI) is a summary measure of countries’ long-term progress in three basic dimensions of human development: income, education level, and health. The HDI intends to be a synthetic measure that, despite expanding the perspective on human development, extrapolates the dimension of economic growth, adding criteria related to the development of individuals: a long and healthy life with access to knowledge and decent living conditions [15].

### Data analysis

We performed a three-step analysis. First, we plotted age-adjusted mortality rate and its 95% CIs. We performed visual inspection of mortality rate time trends for each country and sub-region using a heatmap [16].

Second, we performed a time series analysis. In the temporal trend analysis, we verified whether there was a change in the trend over time, using segmented regression (Joinpoint regression) to identify significant changes over the period [17]. This model assumes a linear trend between the inflection points (joinpoints). In this way, whenever there is a substantial change between a junction of points, it is considered an inflection point and, from this point, a new regression line is started. Joinpoint regression is a data-driven method. This means that it provided potential points in time when the trend changed. From there, these clues help raise hypotheses about what caused the change. In addition, it allows the comparison of different locations to verify whether the changes co-occur or whether there is a time lag between the locations.

The joinpoint regression model, which is composed of a few continuous linear phases, is often used to describe changes in trend data. We define the joinpoint model for the observations, (x_1_, y_1_), …, (x_n_, y_n_), where x_1_ ≤ ⋯ ≤ x_n_, as follows:

$$E[y|x]=\beta_{0}+\beta_{1}x+\delta_{1}{\left(x-\tau_{1}\right)}^{+}+\cdots +\delta_{k}{\left(x-\tau_{k}\right)}^{+}$$

where the τ_k_′s are the unknown joinpoints and a^+^ = a for a > 0 and 0 otherwise

One of the advantages of this method is being able to identify the number and location of changes in the trend, and estimate the annual percentage change (Annual Percentage Change—APC) for each period between inflection points [17].

To estimate the APC, we use the following model:

$$\log(Y_{x})=\beta_{0}+\beta_{1}x$$

where log(Y_*x*_) is the natural logarithm of the rate in year x.

The APC from year x to year x + 1 is:

$$APC=\frac{e^{\beta_{0}+\beta_{1}(x+1)}-e^{\beta_{0}+\beta_{1}x}}{e^{\beta_{0}+\beta_{1}x}}x100=(e^{\beta_{1}}-1)x100$$

An approximate 95% confidence interval for the APC is (APC_L_, APC_U_), where:

$${APC}_{L}=\left(e^{log(APC+1)-1,96\sqrt{w_{x}^{2}\sigma_{x}^{2}})}-1\right)\;x100;\;{APC}_{U}=\left(e^{log(APC+1)+1,96\sqrt{w_{x}^{2}\sigma_{x}^{2}})}-1\right)\;x100$$

considering σ^2^_x_ as the estimate of the variance of bx obtained from the fit of the joinpoint model.

We obtained the number of inflection points through a permutation test through a Monte Carlo resampling. Once we have defined the number k of joining points, we compare the different models with k joining points by estimating their Bayesian Information Criterion (BIC).

To eliminate the autocorrelation between the terms of the regression equation, we used variable time centralized in the year in the middle of the time series (2005). Also, to account for homoscedasticity assumption, we used Poisson distribution parameters with robust variance. The regression was adjusted considering the age-adjusted mortality rate as the dependent variable and the "year-centered" independent variable. Selection of the number of inflection points was performed automatically by the Joinpoint Regression Program 4.9.1.0 through Monte Carlo permutation tests. We considered a significance level of 5%, and for the CIs, we adopted a 95% confidence interval.

Third, assuming heterogeneity across countries, we performed a correlation analysis between the countries’ HDI and the age-adjusted mortality rates in the initial and final years of the time series (1990 and 2019, respectively). Initially, we performed the linear correlation for all countries. We detected that the HDI of the countries also changed in the period. It occurred with different intensities, which indicates that the progression of development took place differently across each country. Then, we divided the countries into terciles, and we performed the correlation separately for each tercile group. We used Spearman’s correlation coefficient, for which there is no assumption of normal distribution of variables. We completed the analysis using R 4.1.0.

## Results

### Mortality patterns and trends

Globally, adjusted colorectal cancer mortality rates declined by 4.37% between 1990 and 2019. The pattern of the decline occurred only in the high SDI group (-23.07%). In all other social development clusters, there was an increase. Furthermore, there is a growth gradient of increase for the high-middle, middle, and low-middle SDI groups (respectively 1%, 36.63%, and 39.64%). The low SDI group showed an increase of 17.84%. This description suggests an association effect between SDI and CRC mortality. However, we found a break in the association gradient with low SDI countries. It seems to be related to the timing of the cancer transition in these countries, which possibly have a higher burden of cancers associated with infectious agents, such as gastric and cervical cancer.

All Latin American subregions showed increased CRC mortality rates between 1990 and 2019. However, the magnitudes were different. Andean and Central Latin America showed a similar increase to the low-middle SDI cluster, while southern Latin America showed a similar pattern to the high-middle SDI cluster. We emphasize that geographical criteria form the subregions of Latin America, and the spatial distribution does not necessarily describe the socioeconomic distribution of the countries.

**Fig 1:**
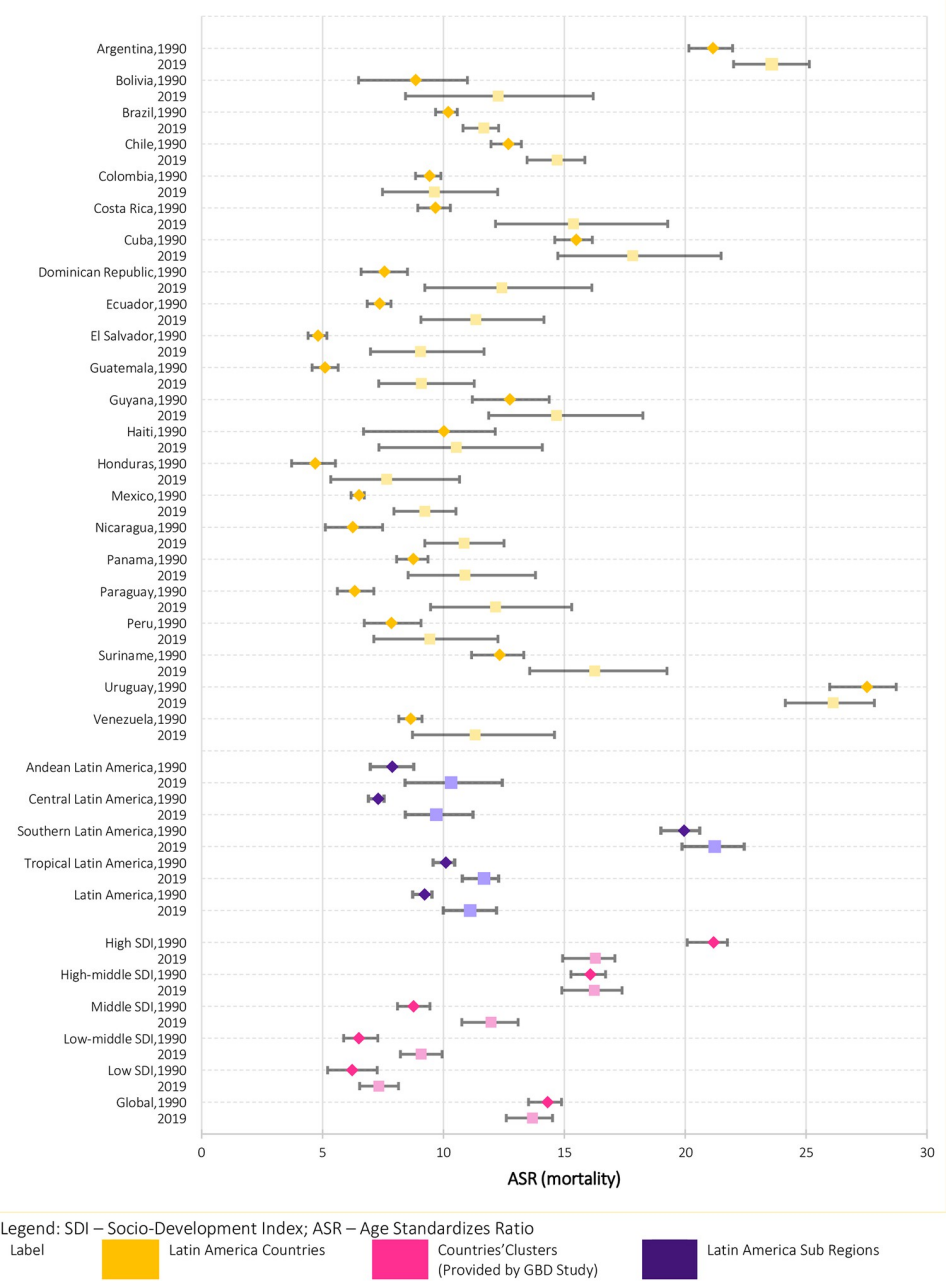
Age-adjusted mortality rates and 95% confidence intervals among Latin American countries, subregions and SDI countries clusters, 1990 and 2019. **Source:** GBD study, 2022. SDI–Socio-Development Index; ASR–age-adjusted mortality rate.

Concerning the countries of the subcontinent, all countries showed an increase in age-adjusted mortality rates between 1990 and 2019 (Fig 1).

Conversely, Uruguay reduced mortality rates, following the high SDI cluster pattern. We found the highest increases between 1990 and 2019 in Paraguay (91.95%), El Salvador (87.94%), and Guatemala (77.95%). The lowest increases occurred in Colombia (2.12%), Haiti (5.03%), and Argentina (11.49%). Uruguay and Argentina had the highest colorectal cancer mortality rates in Latin America and the Southern Latin America subregion. Southern Latin America, which includes these two countries, and Chile had rates of 19.95/100,000 and 21.22/100,000 for 1990 and 2019, respectively). We also note that some countries have wide confidence intervals for 2019, possibly due to a greater imprecision of the mortality indicator in these countries.

**Fig 2:**
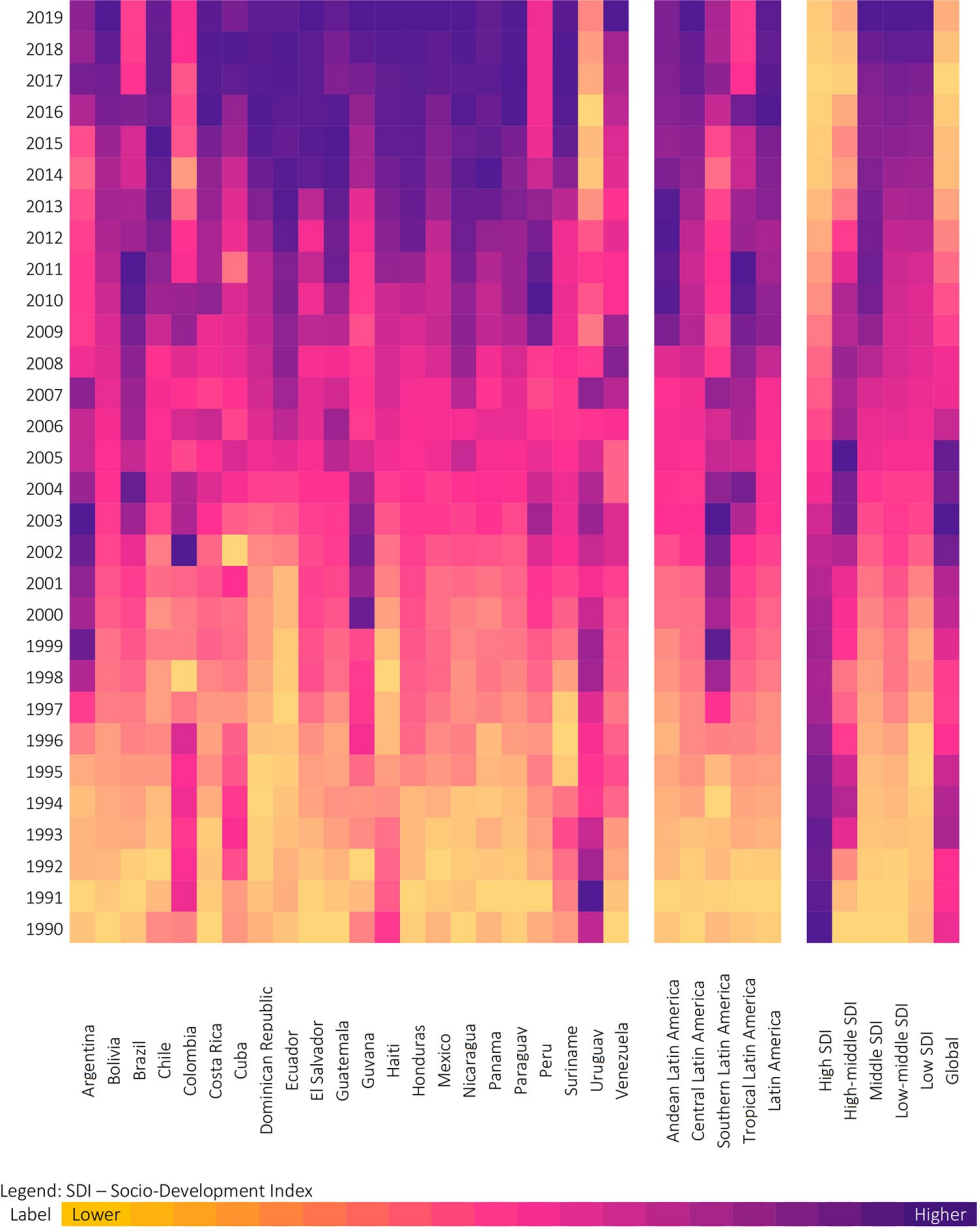
Time series of age-adjusted mortality rate for colorectal cancer among Latin American countries, subregions, and SDI countries clusters, 1990–2019. **Source:** GBD study, 2022. SDI–Socio-Development Index.

From the visual inspection of the trend (Fig 2), we could see more clearly what happened in the last 30 years in Latin America’s SDI clusters and sub-regions. The global trend was upward until the first decade of the 2000s when rates began to fall. This pattern is similar to that observed in the high-middle SDI cluster. In the Middle, low-middle, and low SDI clusters, the tendency was for a gradual increase over the 30 years, with different speeds: the higher the SDI, the faster the growth over the years. Latin America as a whole has similar trend to the Middle SDI cluster. However, analysis shows that Latin America has demonstrated significant heterogeneity among subregions and countries. As we said earlier, Uruguay has a historical series pattern like the high SDI cluster. In contrast, several countries, such as Costa Rica, Dominican Republic, Honduras, Panama, and Paraguay, have a pattern like the low SDI group of gradual and accelerated growth rates of CRC mortality.

The global trend has two distinct periods: one of rising rates up to (APC = 0.02, 95% CI 0.01 to 0.03), followed by a decline up to 2019 (APC = -0.07, 95% CI -0.08 to -0.06). Latin America also has two distinct periods. However, there was an increasing trend in both stages. We could check a deceleration between the first (1990 to 2004, APC = 0.11, 95% CI 0.10 to 0.12) and the second (2004 to 2019, APC = 0.04, 95% CI 0.03 to 0.05).

The SDI clusters were heterogeneous. The high SDI group showed two phases of decline, with a deceleration of the drop. The high-middle SDI group showed a discrete increase phase and a second moment with a reduction. The other groups showed growth in two stages (Middle SDI and Low SDI) or linear growth in a single phase (Low-Middle SDI). Interestingly, there is an apparent equivalence when comparing SDI clusters and Latin American subregions. Andean, Southern, and Tropical Latin America have a trend close to that seen in the High-Middle SDI cluster, while Central America has a similar trend to the Low-Middle SDI cluster.

Moreover, the joinpoint analysis confirmed the disparities in mortality within Latin America (Table 1). Colombia, Costa Rica, Panama, and Paraguay are the only countries with increasing mortality without interruptions. The same trend occurs in Central Latin America and the low-medium-SDI group. Latin America had an average annual increase in colorectal cancer mortality of 0.11% between 1990 and 2004, with slower growth of 0.04% per year between 2005 and 2019. Likewise, most countries and subregions have at least one inflection point. Bolivia, Chile, Cuba, Dominican Republic, Ecuador, Haiti, Honduras, Mexico, and Suriname follow the same trend as Latin America. There is a group whose inflection marks a shift in the mortality rate from a growth phase to a stage of decline: the Andean, Southern and Tropical sub-regions and the high-middle global SDI groups. Argentina, Peru, and Guyana are the only countries with this trend.

**Table 1:** Trends in mortality caused by colorectal cancer in Latin American countries, sub-regions and SDI countries clusters, 1990–2019.

Location	Trend^§^ #1				Trend^§^ #2				Trend^§^ #3				AAPC
	Period	APC	95% CI	p-value	Period	APC	95% CI	p-value	Period	APC	95% CI	p-value	
Argentina	1990–2001	0.29	0.23 to 0.35	<0.001	2001–2014	-0.11	-0.16 to -0.06	<0.001	2014–2019	0.31	0.1 to 0.52	0.009	0.5
Bolivia	1990–2014	0.10	0.09 to 0.11	<0.001	2014–2019	0.21	0.17 to 0.25	<0.001					1.1
Brazil	1990–2003	0.16	0.15 to 0.17	<0.001	2003–2011	0.01	-0.02 to 0.04	0.438	2011–2019	-0.05	-0.08 to -0.02	0.001	0.5
Chile	1990–2008	0.06	0.05 to 0.07	<0.001	2008–2013	0.30	0.16 to 0.44	0.002	2013–2019	-0.03	-0.1 to 0.04	0.489	0.6
Colombia	1990–2019	0.00	-0.01 to 0.01	0.822									0.0
Costa Rica	1990–2019	0.21	0.19 to 0.23	<0.001									1.7
Cuba	1990–2012	0.03	0.01 to 0.05	0.014	2012–2019	0.33	0.22 to 0.44	<0.001					0.6
Dominican Republic	1990–1995	-0.12	-0.30 to 0.06	0.206	1995–2019	0.26	0.24 to 0.28	<0.001					1.8
Ecuador	1990–2000	-0.03	-0.06 to 0.01	0.105	2000–2007	0.51	0.45 to 0.57	<0.001	2007–2019	0.06	0.04 to 0.08	<0.001	1.5
El Salvador	1990–1999	0.31	0.26 to 0.36	<0.001	1999–2019	0.09	0.07 to 0.11	<0.001					2.3
Guatemala	1990–2004	0.25	0.22 to 0.28	<0.001	2004–2019	0.03	0.01 to 0.05	0.011					2.0
Guyana	1990–2002	0.19	0.15 to 0.23	<0.001	2002–2009	-0.17	-0.3 to -0.04	0.017					0,7
Haiti	1990–1998	-0.07	-0.08 to -0.06	<0.001	1998–2005	0.10	0.08 to 0.12	<0.001	2005–2019	0.03	0.03 to 0.03	<0.001	0.2
Honduras	1990–2009	0.09	0.08 to 0.10	<0.001	2009–2012	0.30	-0.09 to 0.69	0.149	2012–2019	0.04	0.01 to 0.07	0.016	1.8
Mexico	1990–2007	0.07	0.06 to 0.08	<0.001	2007–2019	0.15	0.13 to 0.17	<0.001					1.3
Nicaragua	1990–2009	0.23	0.21 to 0.25	<0.001	2009–2019	0.04	-0.01 to 0.09	0.198					1.9
Panama	1990–2019	0.09	0.08 to 0.10	<0.001									0.9
Paraguay	1990–2019	0.23	0.22 to 0.24	<0.001									2.4
Peru	1990–2011	0.12	0.10 to 0.14	<0.001	2011–2019	-0.12	-0.21 to -0.03	0.018					0.6
Suriname	1990–1997	-0.26	-0.47 to -0.05	0.026	1997–2000	0.74	-0.86 to 2.34	0.371	2000–2019	0.13	0.08 to 0.18	<0.001	0.7
Uruguay	1990–2004	-0.01	-0.07 to 0.05	0.823	2004–2019	-0.13	-0.18 to -0.08	<0.001					-0.2
Venezuela	1990–1994	0.28	0.03 to 0.53	0.043	1994–2019	0.05	0.03 to 0.07	<0.001					0.8
Andean	1990–2011	0.14	0.13 to 0.15	<0.001	2011–2019	-0.02	-0.07 to 0.03	0.415					1.0
Central	1990–2019	0.08	0.08 to 0.08	<0.001									1.0
Southern	1990–2001	0.18	0.13 to 0.23	<0.001	2001–2014	-0.07	-0.11 to -0.03	0.002	2014–2019	0.18	0.02 to 0.34	0.041	0.3
Tropical	1990–2004	0.16	0.15 to 0.17	<0.001	2004–2019	-0.02	-0.03 to -0.01	0.009					0.6
Latin America	1990–2004	0.11	0.1 to 0.12	<0.001	2004–2019	0.04	0.03 to 0.05	<0.001					0.7
High SDI	1990–2002	-0.11	-0.12 to -0.1	<0.001	2002–2014	-0.30	-0.32 to -0.28	<0.001	2014–2019	-0.01	-0.06 to 0.04	0.739	-0.9
High-middle SDI	1990–2005	0.07	0.05 to 0.09	<0.001	2005–2019	-0.09	-0.12 to -0.06	<0.001					-0.1
Middle SDI	1990–1996	0.07	0.02 to 0.12	<0.001	1996–2009	0.19	0.17 to 0.21	<0.001	2009–2019	0.01	-0.01 to 0.03	0.448	1.0
Low-middle SDI	1990–2019	0.09	0.09 to 0.09	<0.001									1.2
Low SDI	1990–2000	0.01	-0.01 to 0.03	0.332	2000–2019	0.05	0.05 to 0.05	<0.001					0.6
Global	1990–2004	0.02	0.01 to 0.03	0.016	2004–2019	-0.07	-0.08 to -0.06	<0.001					-0.1

^**§**^ Trends—the number of segments is determined by the number of turning points. When there is one turning point, there are 2-time segments, each with a different trend (in direction and speed); APC—annual percentage change. When the value is negative, AAPC–Average Annual Percentage Change; it indicates a decreasing trend; when it is positive, it means an increasing trend; CI = confidence interval.

**Source:** GBD study, 2022.

The Average Annual Percentage Change (AAPC) analysis summarizes the trend across countries. Except for Uruguay, all Latin American countries had an average increase in mortality rates over 30 years. The same occurred in the sub-regions of Latin America. We recognize, however, a differentiated pattern in the SDI strata: there is an increase for the low, medium-low, and medium SDI strata. The medium-high and high SDI strata, on the other hand, showed a reduction, following the global trend.

Furthermore, we see that there is a parabolic trend: there is an increase between Low-SDI and Low-Middle SDI. Between Low-Middle SDI and Middle-SDI, there is a slowdown in growth. The transition from Middle-SDI to Middle-High SDI changes the trend, now in decline. Finally, between Middle-High SDI and High-SDI, there is an increase in the fall pace. It aligns with our hypothesis that there is a relationship between trends in CRC mortality and development.

### HDI distribution among countries and association with mortality level

Comparison of the trend of CRC mortality rates in GBD SDI clusters, sub-regions of Latin America, and their countries suggests a relationship between temporal trend and aspects of socioeconomic development. That said, we consider it appropriate to estimate the correlation between the adjusted rates and the countries’ HDI. Even though the HDI does not express inequality and economic development patterns, we assume it is a proxy for social development.

Concerning the CRC mortality rate, Latin America grew by 20.56% in magnitude between 1990 and 2019. Most countries showed growth in rates higher than that of the region. The only countries with an increase lower than the Latin American average were Argentina (11.49%), Chile (14.36%), Colombia (2.12%), Cuba (15.02%), Guyana (15.10%), and Haiti (5.03%). Uruguay, as mentioned earlier, had a decline in the period (-5.10%). Regarding the level of human development, we observe that in 1990 most countries were part of the low and medium development groups. In that year, no country belonged to the very high level of the human development group. However, in 2019, most countries moved to the high human development group, with Argentina, Chile, Costa Rica, Panama, and Uruguay classified as having a very high level. The heterogeneity in human development can also be seen in the distribution of mortality rates between countries (Table 2). In the same table, regarding age-adjusted mortality rates, yellow arrows indicate values in countries above Latin America overall, and pink arrows indicate countries with rates lower than the overall rate for the region.

**Table 2:** Changes in HDI and ASR mortality of colorectal cancer among Latin American countries, 1990–2019.

Location	1990				2019			
	HDI		Mortality		HDI		Mortality	
	Index	Category	ASR	Country vs. LA	Index	Category	ASR	Country vs. LA
Argentina	0_._718	High	21.15	**↑**	0.845	Very High	23.59	**↑**
Bolivia	0.551	Medium	8.85	**↓**	0.718	High	12.26	**↑**
Brazil	0.613	Medium	10.21	**↑**	0.765	High	11.67	**↑**
Chile	0.706	High	12.69	**↑**	0.851	Very High	14.71	**↑**
Colombia	0.603	Medium	9.43	**↑**	0.767	High	9.63	**↓**
Costa Rica	0.665	Medium	9.67	**↑**	0.81	Very High	15.38	**↑**
Cuba	0.68	Medium	15.50	**↑**	0.783	High	17.83	**↑**
Dominican Republic	0.599	Medium	7.56	**↓**	0.756	High	12.42	**↑**
Ecuador	0.648	Medium	7.37	**↓**	0.759	High	11.34	**↑**
El Salvador	0.536	Low	4.82	**↓**	0.673	Medium	9.05	**↓**
Guatemala	0.481	Low	5.11	**↓**	0.663	Medium	9.09	**↓**
Guyana	0.548	Low	12.75	**↑**	0.682	Medium	14.68	**↑**
Haiti	0.414	Low	10.03	**↑**	0.51	Low	10.53	**↓**
Honduras	0.519	Low	4.70	**↓**	0.634	Medium	7.65	**↓**
Mexico	0.656	Medium	6.52	**↓**	0.779	High	9.24	**↓**
Nicaragua	0.497	Low	6.25	**↓**	0.66	Medium	10.86	**↓**
Panama	0.675	Medium	8.76	**↑**	0.815	Very High	10.90	**↓**
Paraguay	0.598	Medium	6.34	**↓**	0.728	High	12.16	**↑**
Peru	0.613	Medium	7.85	**↓**	0.777	High	9.44	**↓**
Suriname	*	*	12.33	**↑**	0.738	High	16.26	**↑**
Uruguay	0.694	Medium	27.53	**↑**	0.817	Very High	26.12	**↑**
Venezuela	0.644	Medium	8.65	**↓**	0.711	High	11.31	**↑**
**Latin America**			**9.22**				**11.11**	

HDI–Human Development Index; ASR–age-adjusted mortality rate; LA–Latin America; * not available. ↑ higher than Latina America ASR ↓ Lower than Latin America ASR

**Source:** GBD study, 2022; PNUD, 2021

**Fig 3:**
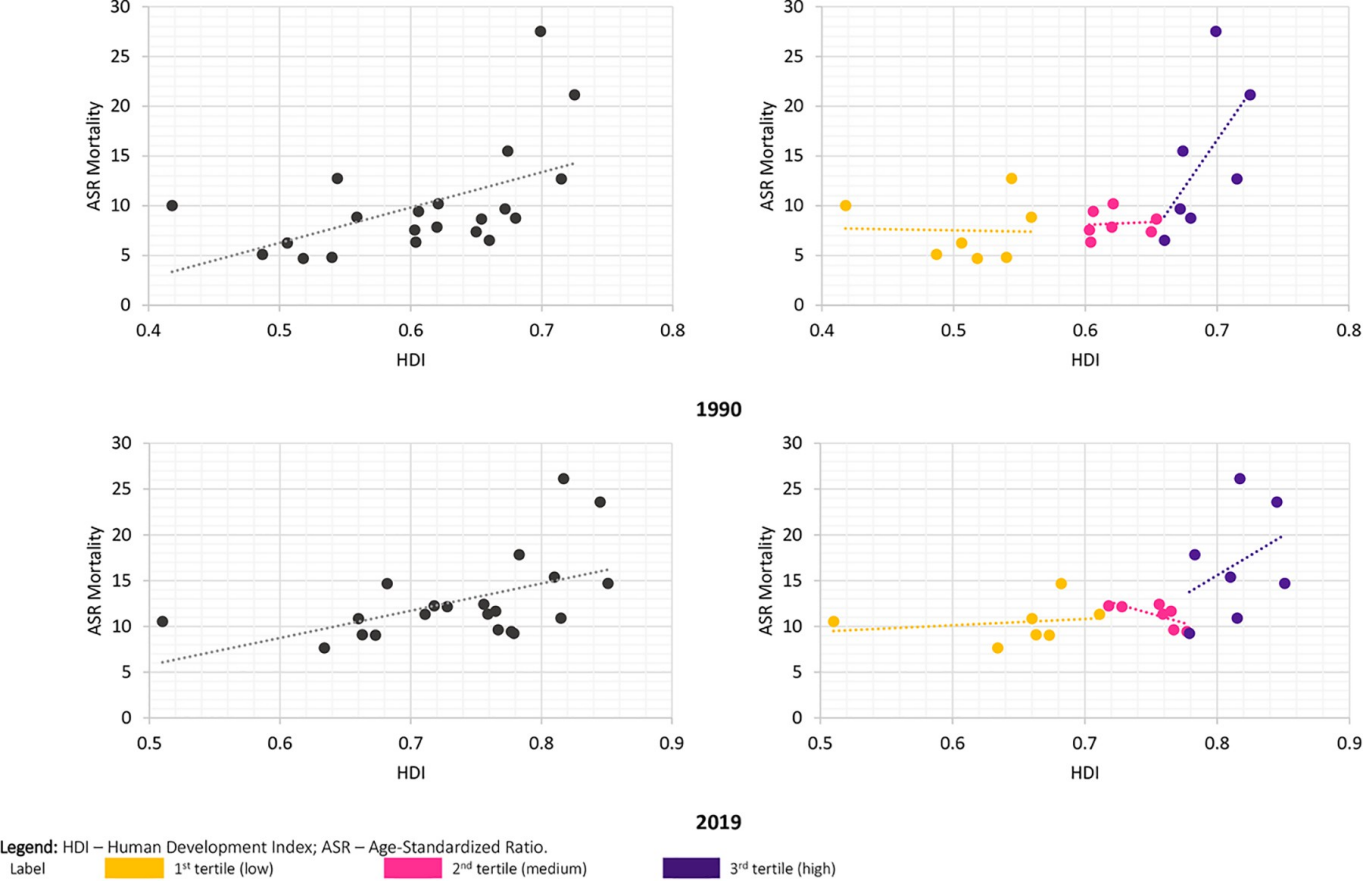
Correlation between HDI and ASR mortality for colorectal cancer among Latin American countries, total and according to HDI group, 1990 and 2019. **Source:** GBD study, 2022; PNUD, 2021. HDI–Human Development Index; ASR–age-adjusted mortality rate.

Finally, the linear correlation between HDI and colorectal cancer mortality rate in the countries is positive regardless of the analysis year (Fig 3). The total correlation strength was similar in 1990 and 2019 (ρ_1990_ = 0.52 vs. ρ_2019_ = 0.51, respectively). The analysis of the groups shows a very different pattern. In 1990, the group with the lowest HDI (Tertile 1) showed a negative correlation (ρ_1990_ = -0.03). The intermediate (Tertile 2) and higher (Tertile 3) HDI groups showed a positive correlation (respectively, ρ_1990_ = 0.09 and ρ_2019_ = 0.62). Only the group with the highest HDI correlation was significant (p = 0.001), although the gradient of association between the groups is notable. In 2019, we observed a positive correlation for the group with the lowest HDI (ρ_2019_ = 0.27; p value = 0.034), a negative correlation for the intermediate HDI group (ρ_2019_ = 0.50; p value = 0.003), and a positive correlation for the group with the highest HDI (ρ_2019_ = 0.38; p value = 0.008). All correlations were significant.

## Discussion

Colorectal cancer mortality rates in Latin America were shown to increase from 1990 to 2019. However, mortality trends between Latin Americans sub-region and countries indicate significant heterogeneity. The differences in colorectal mortality trends between the subregions and countries, both regarding the magnitude and direction of the rates, reflect changes over time coinciding with shifts in human and economic development. Consequently, these findings extend evidence suggesting that socio-economic inequalities status acts as one of the main factors of premature mortality from cancer even on a global scale [18].

Although there has been some economic and social progress in recent years, Latin America is still among the regions with greatest inequality on the planet. The United Nations describes that the wealthiest 10% in Latin America concentrate 37% of income in the region, while the poorest 40% represent only 13% of income. There was, in fact, an increase in the middle-income strata in the area. Even so, the poorest classes continue to experience different needs and vulnerabilities [19]. Social and political scientists attribute this to the region’s colonial history, contributing to a pattern structural heterogeneity that involves the social formation of countries, racism, and secular gender inequities in the region. We highlight that a high level of economic inequality creates political systems that help perpetuate this economy, which is possibly why this heterogeneity persists over time [20].

The study by Carioli et al. [21] estimated cancer mortality statistics for the seven large Latin American countries, focusing on colorectal cancer. Although colorectal cancer mortality was relatively low, the authors noted the persistence of marked variability in rates across Latin American countries. These findings reflect the lifestyle of the region’s population, including a diet rich in red meat and processed foods [22] with unfavorable impacts on traditional demographic and epidemiological indicators, such as life expectancy [23] or causes of death [24].

Another contributing factor to the observed mortality trends is the i-aging population, as the large contingent has a greater proportion of older individuals at that contribute to the burden of colorectal cancer in the region [25]. The burden of disease is also a result of cohort and age effects, suggesting that younger generations benefit from not maintaining risky habits. In addition, we note the different strategies adopted by regions for the diagnosis and screening of colorectal cancer, and the differences seem to influence the variation observed between countries. Few Latin American countries have developed guidelines for the early detection of colorectal cancer, and adherence is quite variable [26].

### Inequalities matter

Inequality influences different dimensions of colorectal cancer. Based on the Theory of Fundamental Causes, postulated by Phelan & Link [27], it can be said that social inequalities have an implied relationship with colorectal cancer. On the one hand, social conditions strongly influence exposure to some risk factors of colorectal cancer [28]. On the other hand, these disparities restrict people’s access and use of health services. They are a by-product of the low living conditions in deprivation. Therefore, greater vulnerability determines the late diagnosis and inadequate access to treatment and postoperative care. Although there is significant variability in survival among countries with similar incomes, it is systematically lower in the poorest countries [29]. Because of this, there is a consistent association between deprivation and excess mortality and survival from colorectal cancer.

Countries undergoing rapid social and economic changes show rapid increases in cancers that are already more frequent in high-income countries [30]. Therefore, colorectal cancer is considered one of the most apparent markers of the cancer transition. In countries in great transition, incidence rates tend to increase uniformly with the increase in the HDI. The explanation for this is not trivial. This location has a fraction of 53.2% attributable to smoking, obesity, diet and physical inactivity [31]. Possibly this relationship can be explained by changes in lifestyle factors and diet, towards a greater intake of animal foods, red meat and processed foods and a more sedentary lifestyle, leading to a decrease in physical activity and increased prevalence of overweight and obesity [32]. On the other hand, the decline in colorectal cancer incidence in some high-incidence countries has been attributed to population-level shifts towards healthier lifestyle choices and the adoption of screening. Therefore, there is a turning point in this association between HDI and colorectal cancer mortality.

Although GBD data are widely explored worldwide, we did not find any that seek to discuss in the light of a specific region, as is the case of Latin America. Indeed, it is a block with many peculiarities regarding socioeconomic inequalities and structural disparities. That said, we consider it reasonable to highlight the differences between countries so that this evidence can be used in discussions by international organizations, such as PAHO, to study the adoption of recommendations for the entire block of countries, following the example of what do with some neglected diseases and violet causes. We consider it appropriate to remember that colorectal cancer, as we mentioned, is the most prominent cancer in the global canary due to the significant increase it has been showing in the last decade. Additionally, adopting these recommendations may come together with multilateral cooperation between countries, considering the potential some countries have in their universal systems, as is the case of Brazil, and others in which social development secretariats endorse health recommendations, as is the case in Uruguay. The situation in Latin America, therefore, makes the dynamics of the disease in this block unique.

### A statistical paradox rises

Socio-economic indicators are independent predictors for all stages of the natural history of cancer [33]. Specifically, the HDI is often inversely associated with cancer-related events (incidence and mortality). When considering cancer types separately, this relationship is relative. Initially, there was a positive relationship between the increase in HDI and colorectal cancer mortality, well documented in the literature [34]. However, variations within each HDI level are evident. The study showed, more recently, that for colorectal cancer, the variation between countries within an HDI level appears to increase from low HDI to very high HDI, with mortality rates more dispersed among nations with higher HDI [35]. Therefore, there are risk variations within HDI levels. On the one hand, it results from the transition of human development, which creates a differential in access to health services.

On the other hand, it differentiates exposure to known risk factors, such as diet and physical inactivity. Therefore, the data suggest an inflection point in the HDI for which access to ultra-processed foods is not encouraged, and healthier habits are encouraged [36]. There is a reduction in exposure to the leading risk factor, and the incidence and mortality from colorectal cancer tend to decrease.

The mechanism underlying the change between human development and colorectal cancer supports the cancer transition theory [37]. According to this theory, as nations develop, they experience decreasing infection-associated cancers and an increasing burden of lifestyle-related cancers. The extension of this theory suggests that, at some point, there is also a reduction in lifestyle-related cancers, by an overall improvement in people’s quality of life to an optimal level when people start to die of natural causes. It is a very advanced phase that is currently being experienced by clusters in very high HDI-developed countries.

The variation around this association can affect the data known as Simpson’s Paradox. It is an extreme confounding condition where an apparent association between two variables is reversed when data are analyzed within each stratum of a confounding variable [38]. This phenomenon has long been recognized as a theoretical possibility, but few real examples have been presented. We observed for Latin American countries that, depending on the HDI stratum to which a country belongs, the association between HDI and mortality can be reversed or remain without statistical significance. For all countries, the association is positive and statistically significant. The relationship with colorectal cancer is an example, possibly because exposure to factors associated with higher cancer occurrence (diet and physical inactivity) [31] and worst prognosis (access to diagnostic and therapeutic services) [8] differ among these groups. It confirms the idea that the socio-economic context has a mediating effect on exposure and outcome relationships for some causes of death.

In any case, the comparison between the pattern of the Latin American Region, its sub-regions and the groups obtained from the social development index allowed us to corroborate the hypothesis that the HDI is an important marker of the transition from cancer [8, 26] and acts as an effect modifier according to development level. This phenomenon can be better observed in Latin America precisely because of the significant disparity found in the subcontinent.

Simpson’s paradox found in the data is a not-so-common phenomenon. In the case of mortality from colorectal cancer, risk behaviors are presented differently between classes and social groups, directly impacting access to diagnosis and therapy. The description of the phenomenon itself is already an exciting aspect [38]. Furthermore, the meaning of this paradox challenges public health to create strategies differentiated by class and intervention complexity so that cancer control policies are equitable. It is important to remember that the comparison was made across countries, but this heterogeneity is also observed at the subnational level. For this reason, the results can be helpful both for multilateralism and for internal measures in each country.

### Strengths and limitations

The study has limitations. First, countries have very different quality of death data and accurate population estimates that allow the calculation of crude and adjusted mortality rates. We emphasize that the method used by the GBD study to make the databases compatible is robust and minimizes information bias. In addition, the study carried out an analysis by Country, and it is unable to detect the great inequality at the subnational level in these countries, as has already been observed for colorectal cancer in Brazil [39], Uruguay [40] and Argentina [41].

Second, the study focuses mainly on mortality. Although the incidence/mortality ratio is a pivotal factor in making fair comparisons between countries, the study aimed to analyze mortality data available and adjusted to the geographic scale analyzed. Cancer incidence data (not just colorectal cancer) has numerous restrictions at the national and sub-national levels, as is the case in Brazil, for which hospital cancer registries and population-based cancer registries have limited coverage [42]. Indeed, one way to study incidence in this situation is to estimate incidence ratios from mortality data with a specific method [43]. However, the incidence/mortality relationship is not necessarily direct, especially in cancer sites with more significant survival potential, such as colorectal cancer. We believe that estimating the incidence from mortality data (the methodology most used today in countries with low capacity for cancer registries) can artificially point out wrong associations. In addition, we reinforce that mortality is an extreme event in the natural history of the disease. It describes well not only exposure to risk factors that determine the occurrence of the disease but also timely access to diagnosis and treatment. Therefore, we consider that mortality is an adequate proxy for the natural history of the disease and for which the association with inequality can be analyzed.

Third, the comparison is made between countries and their HDI. The HDI of a country is the reflection of the socio-economic structure. The actual difference in cancer outcomes is more likely due to differences in the socio-economic level of people that conditions their access to quality health services. Indeed, the HDI has limitations. First, it is a synthetic indicator, which can mask the weight of one dimension to the detriment of the other. Second, there are substantial differences at the subnational level [44]. However, the HDI is a universally known indicator, making it easier to read about its interpretation. Even though differences in cancer outcomes occur by socio-economic level, the HDI has even been adopted by the International Agency for Research on Cancer (IARC) to assess the relationship between cancer locations and development and inequality [45] in the absence of a more robust indicator of global coverage. Because it includes income-related issues. It also considers aspects of education—which is viewed as a proxy for knowledge, attitudes, and preventive health practices—and longevity, a known independent risk factor for several cancer sites, including colorectal cancer.

## Conclusions

The study of cancer and its demographic distribution reflects the living conditions of populations and the development of society. Socio-economic inequalities in treatment can occur for some types of cancer, and colorectal cancer is one of the most sensitive to health inequities.

The relationship between these two phenomena–cancer and human development–extends to exposure to known risk factors for colorectal cancer and diagnostic and therapeutic opportunities. Therefore, recognizing regional inequalities caused by differences in development is essential to decentralize actions to become more effective, considering social disparities to ensure equity in the management of health policies. In this way, including the socio-economic context and its setbacks, such as inequality, is necessary for adequate monitoring and the provision of preventive, diagnostic and curative services to reduce the burden of disease globally.
